# The role of early life genistein exposures in modifying breast cancer risk

**DOI:** 10.1038/sj.bjc.6604321

**Published:** 2008-04-08

**Authors:** A Warri, N M Saarinen, S Makela, L Hilakivi-Clarke

**Affiliations:** 1 Functional Foods Forum, University of Turku, Turku, Finland; 2 Department of Oncology, Lombardi Cancer Center and Georgetown University, 3970 Reservoir Road, NW, Washington, DC 20057, USA; 3 Department of Biochemistry and Food Chemistry, University of Turku, Turku, Finland

**Keywords:** breast cancer, tumour suppressors, epigenetic, mammary stem cell, genistein, soy

## Abstract

Review of the existing literature suggests that consumption of soy foods or an exposure to a soy isoflavone genistein during childhood and adolescence in women, and before puberty onset in animals, reduces later mammary cancer risk. In animal studies, an exposure that is limited to the fetal period or adult life does not appear to have the same protective effect. A meta-analysis of human studies indicates a modest reduction in pre- and postmenopausal risk when dietary intakes are assessed during adult life. These findings concur with emerging evidence indicating that timing may be vitally important in determining the effects of various dietary exposures on the susceptibility to develop breast cancer. In this review, we address the mechanisms that might mediate the effects of an early life exposure to genistein on the mammary gland. The focus is on changes in gene expression, such as those involving BRCA1 and PTEN. It will be debated whether mammary stem cells are the targets of genistein-induced alterations and also whether the alterations are epigenetic. We propose that the effects on mammary gland morphology and signalling pathways induced by pubertal exposure to genistein mimic those induced by the oestrogenic environment of early first pregnancy.

## GENISTEIN AND BREAST CANCER

The assumption that high soy intake among Asian women explains their low breast cancer risk has led to numerous studies carried out in human populations and animal models. Human studies suggest that there indeed is a modest inverse association between high soy food consumption and reduced risk (Trock *et al*, 2006; Wu *et al*, 2008). However, several questions remain regarding a causative role of soy consumption in reducing breast cancer risk; the possibility that the association simply reflects other factors/‘healthy’ lifestyle affecting breast cancer risk cannot be excluded at the present time.

Many animal studies do not support an association between an exposure to this phytochemical during adulthood and reduced mammary tumorigenesis (Tables 1 and 2). The protective role of soy intake during adulthood is further questioned by the results obtained in an intervention study on premenopausal women showing that exposure to 100 mg isoflavone (IF) supplement per day for 1 year did not reduce mammographic density (Maskarinec *et al*, 2003). Mammographic density is a biomarker of increased breast cancer risk; high density increases the risk by four- to six-fold. Further, the consumption of soy foods containing approximately 50 mg IF per day for 2 years did not affect circulating steroid hormones or sex hormone-binding protein (Maskarinec *et al*, 2004), and thus the proposed reduction in breast cancer risk by soy cannot be explained through its effects on serum hormone levels in adults.

One possible explanation for the discrepancy between the protective role of soy found in two meta-analyses (Trock *et al*, 2006; Wu *et al*, 2008) and the lack of effect on biomarkers of breast cancer risk or the results generated in animal studies during adult exposure to soy/genistein is that, to be protective, this bioactive food component may need to be consumed during early life. In support of this argument, epidemiological studies indicate that childhood/adolescence exposure to soy provides protection against breast cancer later in life (Shu *et al*, 2001; Wu *et al*, 2002). Animal studies are in agreement with the findings obtained in humans, and we will briefly review the data on the effects of genistein/soy exposure *in utero* and/or during prepuberty on mammary tumorigenesis in animals. Studies carried out in rats and mice are discussed separately because the tumorigenesis models used in the two species are different.

### Rats

Several studies have examined whether an exposure to genistein or soy protein isolate (SPI), either *in utero* or prepubertally, or a combination of both, affects later mammary tumorigenesis. In addition to genistin (the glucoside conjugate of genistein) and genistein (the aglycone), SPI contains daidzin (the glucoside conjugate) and daidzein (the aglycone), the other main IFs in soy. Daidzein has weaker oestrogenic properties than genistein and generally has not been considered responsible for the biological actions of soy. The third IF of SPI is glycitin and its aglycone glycitein, but they are present only at low levels.

As shown in Table 1, the findings obtained in rats exposed to SPI or purified genistein during the fetal-perinatal period vary from study to study, with three studies reporting an increase in the incidence of either carcinogen-induced tumours (Hilakivi-Clarke *et al*, 1999; Hilakivi-Clarke *et al*, 2002) or spontaneous hyperplasias and ductal carcinomas *in situ* (Foster *et al*, 2004), one study reporting a reduction in tumour multiplicity and % high grade tumours (Su *et al*, 2007a), and two studies reporting no effects (Lamartiniere *et al*, 2002; Pei *et al*, 2003). Two of these studies investigated the effects on mammary gland morphology in adult rats (Hilakivi-Clarke *et al*, 2002; Foster *et al*, 2004). In one study, genistein increased the number of terminal end buds (TEBs) (Hilakivi-Clarke *et al*, 2002), and the other study showed that genistein induced ductal hyperplasias (Foster *et al*, 2004). TEBs have at least two key functions: (1) they are located at the tips of growing ducts and lead the invasion of the epithelial tree to fill the mammary fat pad; (2) they are the sites where malignant transformation takes place (Hilakivi-Clarke, 2007). TEB number peaks after puberty onset at 1 month of age, and they disappear when the epithelial tree has filled the fat pad at 2 months of age. The corresponding structures in the human mammary gland are terminal ductal lobular units (Cardiff, 1998), of which 90% of human breast cancers originate.

Studies examining whether an exposure to soy/SPI/genistein, which started at conception and continued until weaning, affects mammary tumorigenesis found a reduction in tumour multiplicity (Fritz *et al*, 1998; Table 1). Two groups have examined the effects of SPI exposure, which began during gestation and continued throughout adulthood on mammary tumorigenesis, reporting a reduction in mammary tumour incidence and/or multiplicity (Hakkak *et al*, 2000; Su *et al*, 2007a). Interestingly, the study by Hakkak *et al* (2000) found a decline in tumour incidence only in the second generation. The data regarding prepubertal exposure to genistein are very consistent in showing a reduction in mammary cancer risk. Table 1 indicates that all studies investigating an effect on mammary tumorigenesis report a reduction in risk in rats exposed to genistein via feed or through subcutaneous administration. The effects of genistein exposure on mammary gland morphology have been recently reviewed by Warri *et al* (2007), and they are consistent with the protective effect. All studies that investigated mammary gland morphology at the time the gland is most susceptible to malignant transformation indicate a reduction in the number of TEBs and an increase in lobular differentiation (Table 1).

A human parallel to the early life (neonatal) animal studies exists, infants exposed to soy formula. This exposure parallels an exposure during the first 3 weeks of life in rodents. However, at the present time, it is not known whether the high perinatal soy exposure among infant soy formula users has any effect on later breast cancer risk. The conclusions reached in reviews discussing the safety of infant soy formula suggest that it does not appear to cause adverse general health or reproductive outcomes or affect human growth (Strom *et al*, 2001). More cautious authors propose that the existing data are insufficient to draw definitive conclusions on safety and that the use of soy protein formulae should be limited only to those who cannot consume casein-based formulas (Turck, 2007).

Studies that have investigated whether lifetime exposure that begins at puberty has any effects have generated opposing findings (Table 1). Only one of the five studies that exposed rats to genistein or SPI found any effect on mammary tumour incidence or multiplicity (Constantinou *et al*, 2001), and two studies reported a longer tumour latency (Constantinou *et al*, 2001; Gallo *et al*, 2001). The study of Constantinou *et al* (2001) found that an exposure to SPI that was depleted of IFs had the most significant effect on tumour latency and multiplicity. Also, an exposure to 10% miso was reported to reduce mammary tumorigenesis in rats (Gotoh *et al*, 1998).

To summarise, genistein/SPI exposure limited to the prepubertal period appears to reduce later mammary cancer risk in rats, but the effect is mostly lost if the exposure remains high throughout adult life. It is noteworthy, however, that the genistein/SPI formulations and routes of administration in different studies varied (injected *vs* in the diet), which makes it difficult to draw definitive conclusions by comparing results of these studies. However, although genistein/SPI exposure *in utero* may increase susceptibility to malignant transformation, if the exposure continues to adulthood a reduction in risk may occur. The latter is consistent with low breast cancer risk among Asian women who are exposed to soy throughout their lifetime, suggesting that *in utero* exposure primes the gland for later protective effects of genistein. Why a similar reduction in risk is not seen in rats in which exposure started postnatally and continued through the rest of the study is not clear.

### Mice

Studies on the effects of genistein/soy on mammary tumorigenesis in mouse models are scarce, and all studies but one have used a transgenic mouse model in which tumours are caused by an oncogene expressed in the mammary gland during embryogenesis (Table 2). Further, the mouse studies are more diverse than the rat studies concerning the age when the mammary gland morphology was studied. The findings obtained in these studies show that mice exposed to genistein during fetal development exhibit an increase in the number of TEBs as adults (reviewed by Warri *et al*, 2007). According to different studies, neonatal and/or pubertal genistein/SPI exposure either increases (Luijten *et al*, 2004; Thomsen *et al*, 2005) or reduces (Yang *et al*, 2003) epithelial differentiation.

No studies have examined the effects of exposures to genistein/soy that were limited to the fetal or prepubertal period on mammary tumorigenesis. Only one study in mice has investigated whether an exposure from conception until puberty to soy IFs affects mammary tumorigenesis (Table 2; Luijten *et al*, 2004). The study found that Prevastein®, an IF concentrate, not only increased mammary tumour multiplicity and burden but also accelerated mammary gland development (Luijten *et al*, 2004). This observation is opposite to the findings obtained in rats showing that an exposure from conception to weaning is protective against carcinogen-induced mammary tumours.

There are a total of seven studies carried out in mice that were fed SPI or soy meal from the prepubertal period onwards, until the tumour monitoring was completed (Table 2). The studies reported that the exposure reduced (Rao *et al*, 1997; Liu *et al*, 2005), increased (Thomsen *et al*, 2006), or had no effect (Day *et al*, 2001; Jin and MacDonald, 2002; Luijten *et al*, 2004) on mammary tumour incidence and/or multiplicity and size. In addition, a delayed tumour onset after genistein or soy exposure was reported in two of the six genetically modified mouse studies (Jin and MacDonald, 2002; Yang *et al*, 2003). Thus, the data obtained through studies carried out in mice conflict with each other, and it is not clear whether the diversity in outcome can be explained by differences in the route of administration, doses used, or the type of exposure (genistein *vs* SPI *vs* soy meal). Furthermore, studies carried out using rats and mice differ by the model systems used (carcinogen-induced in rats or oncogene-driven in mice) and the differences in mammary gland morphology and oestrogen responsiveness of the established tumours (responsive in rats or non-responsive in mice), which can explain why the data generated in the rat and mouse studies are diverse. Whether these findings can be explained by the oestrogenicity of genistein remains to be investigated.

## MECHANISMS

Two types of changes in the mammary glands of individuals exposed to genistein early in life may mediate the effects on later cancer risk: alterations in gene expression and morphological changes. It is most likely that both are involved and that they interact with each other; that is, changes in gene expression affect morphology and vice versa. In addition, it is possible that the better bioavailability of IFs in children, compared to adults (Halm *et al*, 2007), affects the findings.

As discussed above, the effects of early life genistein exposures on mammary gland morphology and the developmental stage, when animals were examined, vary from study to study, making it difficult to tie the changes in the morphology to observed or anticipated changes in mammary tumorigenesis. An exception is prepubertal exposure to genistein: all the studies carried out, showing a reduction in mammary tumorigenesis in rats, also report consistent changes in mammary gland morphology. This exposure induces differentiation of the mammary epithelium by eliminating TEBs and increasing the number of differentiated lobular-alveolar structures (Table 1). These morphological changes may be initiated by genistein-induced alterations in the mammary stem cell number and/or fate. Of the various epithelial structures, TEBs are believed to contain the highest number of mammary stem cells (Woodward *et al*, 2005).

### Stem cells

During mammary gland development, many stem and progenitor cells gradually commit to differentiation pathways, but some remain uncommitted or partly differentiated within the mammary tissue (Smalley and Ashworth, 2003). Both the stem cell number and their fate may be determined during critical windows of mammary gland development; that is, during *in utero* period, puberty, and pregnancy. The hormonal environment associated with these stages might affect breast cancer risk by increasing or reducing the total number of replicating stem cells, their lineage-specific differentiation to myoepithelial and luminal cells, and eventually the number of cells at risk for malignant transformation (Trichopoulos *et al*, 2005). There is accumulating evidence for the hypothesis that breast cancer risk is determined in part by the number of susceptible breast stem/progenitor cells that can serve as targets for transformation (Liu *et al*, 2008).

Stem cells have been linked to pregnancy-induced dual effects on breast cancer risk. Although pregnancy, pregnancy hormonal environment, and soy exposures during pregnancy in affecting mother's risk are not discussed in this review, the mechanisms by which pregnancy alters susceptibility to develop breast cancer may be relevant in explaining the protective effects of prepubertal genistein and oestrogen exposures. We (Cabanes *et al*, 2004) and others have shown that peripubertal oestrogen exposure reduces mammary gland tumorigenesis in animal models. According to Sivaraman *et al* (1998), pregnancy hormones induce a molecular switch in mammary stem cells that inhibits cell proliferation in response to subsequent exposure to hormones or carcinogens. Wagner and Smith (2005) have identified cells in the mammary gland that are likely to represent pregnancy-related epithelial cells (PI-MECs) with stem cell characteristics. These investigators have suggested that the PI-MECs, which do not undergo apoptosis during post-lactational remodelling and thus persist the rest of the (mouse's) life after the first pregnancy–lactation cycle, could be the mediators of the long-term pregnancy-related protective effects against breast cancer (Wagner and Smith, 2005). However, in a transgenic mouse model prone to pregnancy-related mammary tumorigenesis (MMTV-neu mice), the PI-MECs may be the targets for neoplastic transformation (Wagner and Smith, 2005), underlining the complexity of mammary stem cells and their role in malignant transformation. Several excellent papers have been published regarding the effects of pregnancy on gene expression, and clearly some of the changes induced by pregnancy and pubertal genistein exposure are similar, such as upregulation of BRCA1 and p53 (Cabanes *et al*, 2004; de Assis and Hilakivi-Clarke, 2006).

### Changes in cell proliferation and apoptosis

If an early life exposure to genistein, or other oestrogenic compounds, alters breast cancer risk by targeting epithelial stem or progenitor cells, changes in cell proliferation and apoptosis are expected to be seen. For example, a higher number of stem cells in individuals exposed to oestrogenic compounds *in utero*, or to diets that increase later mammary cancer risk, might be associated with increased cell proliferation, either by inducing proliferation or by reflecting a need for stem cells to fullfil the demand of increasing the number of luminal and myoepithelial mammary cells to form TEBs, lobules and ducts. An illustrative example is caveolin-1 knockout mice, which exhibit dysregulation of mammary stem cell self-renewal, probably due to increased Wnt/*β*-catenin signalling (Sotgia *et al*, 2005).

Fetal exposure to genistein has been reported to increase mitotic activity in the mammary gland (Foster *et al*, 2004), which is consistent with the findings linking this exposure to increased risk of developing mammary tumours. The effects of prepubertal genistein exposure on cell proliferation in rats are also consistent with the effects on tumorigenesis. When determined using the heavy water labelling method (Kim *et al*, 2007) or by labelling the cells with Brdu (Pei *et al*, 2003), exposure to genistein in the prepubertal period inhibited mammary epithelial cell proliferation. If the genistein exposure continues to adulthood, it may impact neither cell proliferation nor apoptosis (Gallo *et al*, 2002), again in agreement with the findings regarding the effects on mammary tumorigenesis.

### Changes in gene expression

The means by which hormonal changes would affect stem cells might be due to changes in gene expression. As an attempt to define the key changes in mammary reprogramming by hormones, an extensive list of genes attributed to mammary stem cells has been presented (Woodward *et al*, 2005). To summarise, stem cell fate, that is self-renewal and differentiation along specific lineage pathways, is regulated by multiple factors. In the mammary gland, Wnt/*β*-catenin, Notch and Hedgehog signalling pathways impact mammary stem/progenitor cell fate. BRCA1 (Sotgia *et al*, 2005) and phosphatase and tensin homologue deleted on chromosome 10 (PTEN) (Stiles *et al*, 2004) may also be involved. In particular, BRCA1 is suggested to play a critical role in the differentiation of oestrogen receptor (ER)-negative stem/progenitor cells to ER-positive luminal cells (Liu *et al*, 2008). As discussed below, genistein affects the expression of some of the genes that regulate mammary stem cell fate.

Many of these changes in gene expression induced by genistein reflect the chemical structure of this phytochemical and thus the activation of the ERs, but some are due to genistein's ability to inhibit several protein kinases. Interestingly, the effects of genistein on the ER expression are variable between different studies, with some reporting a reduction (Pei *et al*, 2003) and some an increase in the ER-*α* levels (Su *et al*, 2007a). Studies are relatively consistent in showing an upregulation of ER-*β* by genistein. Oestrogen receptors may be linked to stem cells, although it is not clear whether mammary stem cells express or do not express this receptor.

It is difficult to determine the importance of the genes identified in microarray studies in human breast cancer cells in mediating the preventive effects of genistein, as the changes may not have a direct impact on altering the susceptibility to mammary tumorigenesis. A recent microarray study, which used the mammary glands of adult rats exposed to genistein via diet from conception onwards, identified several genes that relate to stem cell fate and/or regulate mammary cell proliferation, apoptosis and differentiation, including increased expression of Sfrp2 (Wnt receptor) and Hes1 (Notch signalling pathway), and reduced expression of Wnt5a and Notch2 (Su *et al*, 2007b). Since this exposure was also reported to lengthen tumour latency and reduce tumour incidence and multiplicity (Su *et al*, 2007a), it is possible that genistein/SPI reduces mammary cancer risk by affecting stem cell fate.

## GENISTEIN AND TUMOUR SUPPRESSOR GENES

It is clear that no single genetic change is responsible for the initiation of all breast cancers, but this disease can have a highly varying genetic background. Alterations in the expression of tumour suppressor genes, either by mutations or directly/indirectly due to epigenetic changes, are particularly critical in affecting breast cancer risk. We will briefly review two tumour suppressor genes whose expression is altered in animals exposed to genistein: they are BRCA1 and PTEN.

### BRCA1

Hereditary mutations in BRCA1 not only increase the risk of breast cancer but also risk for ovarian and prostate cancer. Women who have inherited mutations in the BRCA1 tumour suppressor gene have an approximately 66% likelihood of developing breast cancer by the age of 70 (Friedenreich *et al*, 2001). In sporadic breast cancers, BRCA1 mutations are rare; however, 30–40% of sporadic cancers show reduced expression of BRCA1 (see Ashworth, 2004).

BRCA1 has many biological functions. This protein interacts with RAD51 protein (Ashworth, 2004) and, as a result, BRCA1 is capable of acting as a gatekeeper in maintaining genomic integrity by preventing DNA damage and inducing DNA repair. The C terminus of BRCA1 interacts with several transcription factors and thus can regulate basal transcriptional machinery. The importance of BRCA1 for normal mammary development has been demonstrated through a transgenic mouse model, this model expresses a splice variant of BRCA1, which lacks the N-terminal RING finger domain (MMTV-*BRCA1sv*) (Hoshino *et al*, 2007). The Brca1 mutant mice exhibited enhanced TEB proliferation at puberty and marked mammary lobulo-alveolar development, and they had an increased tumorigenesis and accelerated mortality after a challenge by DMBA.

Several findings indicate a link between genistein and BRCA1, although most of the studies focus either on genistein's direct effects on BRCA1 (*in vitro*) or its effects on BRCA1 in adult animals. *In vitro* studies have shown that physiological doses of genistein (0.5–1.0 *μ*M) upregulate BRCA1 in ER+ human breast cancer cells and in prostate cancer cells (Fan *et al*, 2006). In addition, genistein upregulates the expression of both Brca1 and Brca2 mRNA in adult ovariectomised rats (Vissac-Sabatier *et al*, 2003). However, the findings obtained in mice inoculated with mammary tumour cells obtained from conditional *Brca1*−/− mice, showing that dietary exposure to 750 p.p.m. genistein reduced the size of the tumours by 50%, indicate that genistein can be protective in the absence of functional *Brca1* (Tominaga *et al*, 2007). The potential protective effect of pubertal genistein exposure may be linked to an upregulation of *Brca1*: we noted that this tumour suppressor is upregulated in the mammary glands of rats exposed daily to 50 *μ*g genistein or 10 *μ*g E2 during prepuberty (Cabanes *et al*, 2004). Ongoing studies in our laboratory are determining whether prepubertal exposure to 500 p.p.m. genistein affects mammary tumorigenesis in heterozygous *Brca1*+/− mice.

### Phosphatase and tensin homologue deleted on chromosome 10 (PTEN)

Inactivating mutations or deletions of the PTEN gene are among the most common changes found in human cancers, particularly in prostate and endometrial cancers (Blanco-Aparicio *et al*, 2007). Downregulation in PTEN expression or signalling has also been identified in several other types of cancers, including inherited and sporadic breast cancers. The PTEN protein is a lipid phosphatase and has been suggested to act as a tumour suppressor owing to its inhibition of the PI3K/Akt signalling pathway. A recent microarray analysis, comparing endometrial tissues obtained from *Pten*+/− and wild-type mice, identified ER and its downstream targets as genes that might be linked to the increased endometrial cancer risk (Lian *et al*, 2006). Interestingly, PTEN may be one of the key genes BRCA1 has to repair to prevent malignant transformation: frequent gross PTEN mutations, involving intragenic chromosome breaks, inversions, deletions and microcopy number aberrations are found in BRCA1-deficient tumours (Saal *et al*, 2008).

A study carried out utilising rats found that genistein promotes apoptosis in mammary epithelial cells by inducing PTEN (Dave *et al*, 2005). These changes were accompanied by a decrease in mammary tumorigenesis. Fetal exposure to genistein or SPI at doses that had no effect on mammary tumorigenesis did not affect Pten protein expression or apoptosis in the TEBs of adult rats (Su *et al*, 2007a). However, if the exposure to genistein or SPI continued throughout the postnatal life, PTEN immunostaining and apoptosis were increased in the mammary TEBs (Dave *et al*, 2005).

## EPIGENETICS

The mechanisms responsible for persistent changes in gene expression in the mammary glands of individuals exposed to genistein or other (oestrogenic) compounds early in life are most likely to involve epigenetic modifications. In contrast to mutations or other events that cause alterations in the DNA sequence, epigenetic changes cause alterations in gene transcription; that is they determine whether a gene is expressed or not. Two of the best characterised means of epigenetic modifications are DNA methylation and histone modifications (Tang and Ho, 2007). Epigenetics is now known to be the key process during early development that allows the environment to interact with the genotype, resulting in the observed phenotype. Thus, early life dietary exposures may modify later breast cancer risk through epigenetic changes that alter the pathways that participate in regulating mammary gland development.

Genistein has been reported to affect DNA methylation (Dolinoy *et al*, 2006). Methylation prevents gene expression, while demethylation leads to increased expression of previously methylated genes. Interestingly, genistein might both methylate and demethylate genes. The evidence that genistein induces methylation comes from studies showing that maternal exposure to genistein increases methylation of six cytosine-guanine sites at the viable yellow Agouti mice (Dolinoy *et al*, 2006), increasing the number of Agouti offspring that exhibit pseudoagouti phenotype, which in turn is linked to lowered cancer risk. However, genistein inhibits DNA methyltransferases (Day *et al*, 2002), which catalyse the addition of a methyl group, causing demethylation of the CpG islands. It is not known whether prepubertal genistein exposure might increase or reduce methylation patterns.

## CONCLUSIONS

The evidence obtained both in epidemiological (Shu *et al*, 2001; Wu *et al*, 2002) and animal studies (Tables 1 and 2) suggests that genistein/soy exposure during the period preceding puberty reduces later susceptibility to develop breast cancer. Further, if the exposure occurs during the fetal period, no protection may be seen, but if it continues from conception onwards, genistein/soy may be protective. At the present time, no convincing explanation can be offered as to why the breast cancer risk-reducing effect of genistein/soy might be strongest during childhood and early adolescence. One possibility is that it is linked to increased differentiation of the mammary gland and elimination of targets that are the sites of malignant transformation (i.e., TEBs and mammary stem/progenitor cells). Thus, early genistein/soy exposure might have similar effects on the mammary gland than early pregnancy does. It is well established that early first pregnancy (before 20 years of age) reduces breast cancer risk, while women undergoing their first pregnancy after age 35 are at an increased risk.

The reduction in breast cancer risk induced by early genistein/soy exposure is also likely to reflect changes in the expression of multiple genes. For example, in animal models, prepubertal exposure to genistein causes a persistent upregulation of Brca1 (Cabanes *et al*, 2004) and it might upregulate PTEN (Dave *et al*, 2005). It is not known whether all these changes (or none of them) lead to a reduction in mammary cancer risk. The complexity between genistein/soy, the genes it activates/inhibits, and breast cancer risk are also illustrated by the fact that genistein activates ER-*α*, which in turn interacts with BRCA1 (Fan *et al*, 2006) and PTEN (Lian *et al*, 2006). The causality between the changes in gene expression induced by genistein/soy intake and altered breast cancer risk can be addressed at least partly by using genetically modified mouse models such as *Brca1* or *Pten* knockout mice.

## Figures and Tables

**Table 1:** Studies carried out in rats investigating the effects of early life genistein/SPI/soy exposure on mammary gland morphology and tumorigenesis

**Exposure time**	**Compound/diet, dose, route of administration**	**Effect on mammary gland morphology**	**Effect on carcinogen-induced mammary tumour growth**	**Reference**
In utero *and perinatal exposure*				
GD 15–19	Genistein 1.5 or 30 mg kg^−1^ per day, s.c.	PND 28: no changes in TEB number	MNU (50 mg kg^−1^ on PND 28): no changes in tumour latency and multiplicity by PND 182	Pei *et al* (2003)
GD 15–20	Genistein 20, 100, or 300 *μ*g per rat per day (∼0.1, 0.5, 1.5 mg kg bw^−1^), s.c.	Not studied	DMBA (10 mg per rat ≈50 mg kg^−1^ on PND 45–50): increased tumour incidence. Follow-up until PND 170–200	Hilakivi-Clarke *et al* (1999)
				
GD 0 – PND 0	Genistein 15, 150, or 300 p.p.m. in AIN-93G diet (produces serum levels corresponding to Asians on high soy diet; Note 1)	PND 56: an increase in the number of TEBs, and a decrease in the number of lobules in the highest genistein group	DMBA (10 mg per rat ≈50 mg kg^−1^ on PND 47): no change in tumour latency, but increased tumour incidence on PND 119 in the high genistein group	Hilakivi-Clarke *et al* (2002)
GD 4 – PND 0	Genistein 250 p.p.m. or SPI (gen 216 mg+daid 160 mg per kg) in AIN-93G diet (Note 1)	Not studied	MNU (50 mg kg^−1^ on PND 51): longer tumour latency in both groups, and lower tumour multiplicity and % high-grade tumours in SPI group on PND 149	Su *et al* (2007a)
GD 0–21	Genistein 250 p.p.m. in AIN-76A diet (Note 1)	Not studied	DMBA (80 mg kg^−1^ on PND 50): no effect on tumour multiplicity. Follow-up until PND 230	Lamartiniere *et al* (2002)
PND 2–8	Genistein 10 mg kg bw^−1^ per day, s.c. (pharmacological dose; Note 1)	PND 200: distended mammary glands with secretion and milk production, ductal hyperplasia, microcalcifications, fibrosis, and necrosis	Spontaneous: increased mammary atypical hyperplasias and *in situ* ductal carcinomas of comedo type on PND 200	Foster *et al* (2004)
				
In utero *and pre/peripubertal exposure*				
GD 0 – PND 21	Genistein 25 or 250 p.p.m. in AIN-76A diet (250 p.p.m. produced serum levels of ca. 700 and 1800 pmol ml^−1^ on PND 7 and 21, respectively)	PND 21 and 50: reduced number of TEBs. PND 50: lower number of lobules type I in the higher genistein group	DMBA (80 mg kg^−1^ on PND 50): a dose-dependent decrease in tumour multiplicity by PND 230	Fritz *et al* (1998)
GD 1 – PND 22	Genistein 300 or 800 p.p.m. in chow diet	PND 22: increased ductal branching in the higher genistein group in males	Not studied	You L, Sar M, Bartolucci EJ, McIntyre BS, Sriperumbudur R (2002) Modulation of mammary gland development in prepubertal male rats exposed to genistein and methoxychlor. *Toxicol Sci* **66**: 216–225
GD 4 – PND 21, 33 or 50	20% SPI in AIN-93G diet (Note 2)	PND 50: reduced number of TEBs	Not studied	Rowlands JC, Hakkak R, Ronis MJ, Badger TM (2002) Altered mammary gland differentiation and progesterone receptor expression in rats fed soy and whey proteins. *Toxicol Sci* **70**: 40–45
				
In utero *→ lifetime exposure*				
GD 4*→* PND 149	SPI (gen 216 mg +daidzein 160 mg per kg) in AIN-93G diet	Not studied	MNU (50 mg kg^−1^ on PND 51): longer tumour latency, decreased tumour incidence and multiplicity by PND 149	Su *et al* (2007a)
GD 0 *→* lifetime (for two generations)	SPI=430 mg total isoflavones, including 276 mg genistein and 132 mg daidzein per kg AIN-93G diet	Not studied	DMBA (80 mg kg^−1^ on PND 50: longer tumour latency, and a decline in tumour incidence in the second generation. No difference in tumour multiplicity or volumes by PND 175	Hakkak *et al* (2000)
GD 7 *→* lifetime	Genistein 5, 25, 100, 250, 625, or 1250 p.p.m. in chow diet	PND 50: increased lobular differentiation, but ductal and alveolar hyperplasia in the higher genistein groups	Not studied	Delclos KB, Bucci TJ, Lomax LG, Latendresse JR, Warbritton A, Weis CC, Newbold RR (2001) Effects of dietary genistein exposure during development on male and female CD (Sprague–Dawley) rats. *Reprod Toxicol* **15**: 647–663
				
*Prepubertal exposure*				
PND 1–21	Genistein: 250 p.p.m. in AIN-76A diet	PND 50: reduced number of TEBs and increased number of lobules	DMBA (80 mg kg^−1^ on PND 50): reduced tumour multiplicity by PND 230	Lamartiniere *et al* (2002)
PND 7, 10, 14, 17, 20	Genistein 20 *μ*g per pup per day (ca.1 mg kg bw^−1^) s.c.	PND 183: increased lobular differentiation	DMBA (10 mg per rat ≈50 mg kg^−1^ on PND 45): reduced tumour multiplicity and number of proliferating tumours on PND 171	Hilakivi-Clarke L, Onojafe I, Raygada M, Cho E, Skaar T, Russo I, Clarke R (1999) Prepubertal exposure to zearalenone or genistein reduces mammary tumorigenesis. *Br J Cancer* **80**: 1682–1688
PND 7–20	Genistein 50 *μ*g per pup per day (ca.3.3−1.25 mg kg^−1^ per day) or E2 10 *μ*g per pup per day s.c.	PND 56: genistein reduced number of TEBs and epithelial density, increased number of lobuloalveolar structures	DMBA (studied only in prepubertally E_2_-exposed rats: a significant decrease in tumour incidence compared to control rats)	Cabanes *et al* (2004)
PNDs 16, 18, 20	Genistein 500 mg kg^−1^ per day (in all studies) s.c.	PND 50: reduced number of TEBs, increased number of lobules	DMBA (80 mg kg^−1^ on PND 50): reduced mammary tumour multiplicity or incidence	Cotroneo MS, Wang J, Fritz WA, Eltoum IE, Lamartiniere CA (2002) Genistein action in the prepubertal mammary gland in a chemoprevention model. *Carcinogenesis* **23**: 1467–1474 Brown NM, Wang J, Cotroneo MS, Zhao YX, Lamartiniere CA (1998) Prepubertal genistein treatment modulates TGF-alpha, EGF and EGF-receptor mRNAs and proteins in the rat mammary gland. *Mol Cell Endocrinol* **144**: 149–165 Murrill WB, Brown NM, Zhang JX, Manzolillo PA, Barnes S, Lamartiniere CA (1996) Prepubertal genistein exposure suppresses mammary cancer and enhances gland differentiation in rats. *Carcinogenesis* **17**: 1451–1457
PND 15–19	Genistein 1.5 or 30 mg kg bw^−1^ per day, s.c.	PND 28: no changes in TEB number	MNU (50 mg kg^−1^ on PND 28): decrease in tumour incidence through PND 182 (low-dose genistein)	Pei *et al* (2003)
PND 23,25,27,29	Genistein 50 mg kg bw^−1^ per day, s.c.	PND 30: increase in mammary gland size, and the number of lobules I, but no effect on the TEBs	Not studied	Brown NM, Lamartiniere CA (1995) Xenoestrogens alter mammary gland differentiation and cell proliferation in the rat. *Environ Health Perspect* **103**: 708–713
*Peripubertal exposure*				
PND 31–45	Genistein 375 or 750 p.p.m. in diet	PND 45: no effects on mammary ductal and lobuloalveolar development	Not studied	Santell RC, Chang YC, Nair MG, Helferich WG (1997) Dietary genistein exerts estrogeneic effects upon the uterus, mammary gland and the hypothalamic/pityuitary axis in rats. *J Nutr* **127**: 263–269
				
*Pre- or peripubertal → for lifetime exposure*				
PND 21 *→*ca.218	0.35 or 0.7% soy extract (Soyselect™, contains 12% isoflavones) in AIN-76A diet	Not studied	DMBA (80 mg kg^−1^ on PND 50): longer tumour latency. No effect on the incidence, multiplicity, or median total tumour burden by PND 218	Gallo *et al* (2001)
				
PND 25*→*158	Genistein 800 or 1600 p.p.m. in AIN-76A diet	Not studied	MNU (50 mg kg^−1^ on PND 50): no significant effects by PND 158	Kim H, Hall P, Smith M, Kirk M, Prasain JK, Barnes S, Grubbs C (2004) Chemoprevention by grape seed extract and genistein in carcinogen-induced mammary cancer in rats is diet dependent. *J Nutr* **134**: 3445S–3452S
PND 36 *→* 127	Isoflavones: 30 400 810 p.p.m. in SPI containing diet	Not studied	DMBA (10 mg per rat on PND 50): no significant effects by PND 127	Appelt LC, Reicks MM (1999) Soy induces phase II enzymes but does not inhibit dimethylbenz[a]anthracene-induced carcinogenesis in female rats. *J Nutr* **129**: 1820–1826
PND 43 *→* ca.176 (F-344 rats)	10 or 20% SPI, with and without isoflavones, in AIN-93G (Note 2)	Not studied	MNU (40 mg kg^−1^ on PND 50): no significant effects by PND 176	Cohen LA, Zhao Z, Pittman BSJA (2000) Effect of intact and isoflavone-depleted soy protein on NMU-induced rat mammary tumorigenesis. *Carcinogenesis* **21**: 929–935
PND 43 *→* ca.163	Genistein 200 p.p.m. or 16% SPI with and without isoflavones, in modified AIN-74A diet (Note 2)	Not studied	DMBA (15 mg per rat on PND 50): decreased tumour multiplicity with SPI, but not with genistein. Most significant effect on tumour multiplicity and latency in the SPI-without isoflavones group	Constantinou *et al* (2001)
PND 49 *→* 175 (CD/Crj rats)	10% miso (fermented soybean product) in diet	Not studied	MNU (50 mg per rat on PND 49): significant decrease in incidence and multiplicity by PND 175	Gotoh *et al* (1998)

Abbreviations: bw=body weight; DMBA=dimethylbenz(a)anthracene; GD=gestational day; MNU=*N*-methyl-*n*-nitrosourea; PND=postnatal day; p.p.m.=parts per million (mg per kg); SPI=soy protein isolate; TEB=terminal end bud.

Note 1: Asian population on high soy diet consumes genistein ca. 1–30 mg per day=ca. 0.02–0.55 mg kg^−1^ per day. Native Japanese adults are reported typically to consume 30–40 mg (aglycone units) of isoflavones per day (Wakai *et al* *J Nutr* 1998; 128: 209–213), their plasma levels being ∼300 nmol l^−1^ (Adlercreutz *et al* *Lancet* 1993; 342: 1209–1210). Genistein intake in the Western Europe ca. 0.005 mg kg^−1^ per day. For extrapolation from human to rodent, small animals need ca. 10 times higher concentrations compared to humans, since they are less susceptible to drugs (Wuttke *et al* *Ageing Res Rev* 2007; 6(2): 150–88). Thus, genistein doses 0.1**–**1.5 mg kg^−1^ per day to rodents would be comparable to average daily intake in Asian countries. Doses >10 mg kg^−1^ per day are pharmacological.

Note 2: SPI contains the isoflavones genistein 216 mg kg^−1^, and daidzein 160 mg kg^−1^, and their *β*-glycosides (Hakkak *et al* *Cancer Epidemiol Biomarkers Prev* 2000; 9: 113–117). SPI (10 and 20%) is estimated to contain ca. 50 and 100 times the intake of an average Japanese adult (Cohen *et al* *Carcinogenesis* 2000; 21(5): 929–935).

Note 3: Sprague–Dawley rats were used unless otherwise stated in the first column, under the ‘exposure time’.

**Table 2:** Studies carried out in mice investigating the effects of early life genistein/soy exposure on mammary gland morphology and tumorigenesis^a^

**Mouse strain, exposure time**	**Compound/diet, dose, route of administration**	**Effect on mammary gland morphology**	**Effect on oncogene- or carcinogen-induced mammary tumour growth**	**Reference**
In utero *exposure (no studies)*				
In utero *and prepubertal exposure*				
FVB/N-TgN (MMTV-neu), GD 0 – PND 21–23	‘Prevastein’ containing (1) genistein 6 mg+no daidzein, or (2) genistein 28 mg+daidzein 12 mg, or (3) genistein 89 mg+daidzein 42 mg per kg high fat diet	PND 70: enhanced mammary gland ‘maturation’ (decreased number of TEBs) in the highest dose group	No change in tumour onset. Increase in tumour burden (multiplicity and mass) in the medium and high isoflavone groups. Follow-up until PND 140	Luijten *et al* (2004)
*Prepubertal exposure (no studies)*				
*Pre- or peripubertal → for lifetime exposure*				
*α*ERKO and WT mice (C57Bl/6J × 129SVJ), PND 21 → lifetime	Genistein 1000 p.p.m. in AIN-76A diet	Not studied	MPA-DMBA^b^: no significant effects by PND 238	Day *et al* (2001)
FVB/N-TgN (MMTV/c-neu), PND 25–196	Soy isoflavones (prevastein): 11, 39, 130 mg per kg high fat (Western style) diet	PND 70: increased mammary ductal branching (all doses, PND 42) and decreased number of TEBs in the medium dose group	No change in tumour incidence. Increase in tumour multiplicity and size (highest dose group) by PND 196	Thomsen *et al* (2005)
TG.NK (MMTV/ c-neu), PND 28–210	Open formula (soy, alfaalfa, wheat, oat, corn) NTP-2000 diet *vs* AIN-76A	Not studied	Open formula diet significantly decreased tumour incidence, multiplicity and tumour weight compared to AIN-76A (follow-up until PND 210)	Rao *et al* (1997)
FVB/N-TgN (MMTV/c-neu), PND 28 → lifetime	SPI including genistein 132 mg+daidzein 89 mg per kg high fat (Western style) diet	Not studied	No significant effects by PND 238	Luijten *et al* (2004)
FVB/N-TgN (MMTV/c-neu), starting on PND 28–35 → lifetime	23.4% soy meal, including genistein 214 p.p.m. and daidzein 277 p.p.m. in Purina 5001 diet	PND 175: reduced mammary ductal elongation and branching (descriptive data). Potentiation of the precocious differentiation by E of the mammary gland (descriptive data)	Longer tumour latency. (Necropsy on PNDs 105, 140, 175, 224, 266, 315, and 420)	Yang *et al* (2003)
FVB/N-TgN (MMTV/c-neu), starting on PND 28–35 → lifetime	Soy meal (Purina 5001) diet including genistein 214 mg and daidzein 277 mg per kg diet, or diet with pure genistein 137 mg and daidzein 74 mg per kg diet	Not studied	Decrease in incidence in soy meal group (follow-up until PND 420)	Liu *et al* (2005)
FVB/N-TgN (MMTV/c-neu), starting on PND 42–49 → lifetime	Genistein: 250 mg, or NovaSoy with genistein 250 mg per kg AIN-93G+the mice were mated and allowed one full-term pregnancy and 2 weeks of lactation during the first 3 month of life	Not studied	Longer tumour latency by both genistein and NovaSoy. No changes in tumour incidence or multiplicity by PND 238	Jin and MacDonald (2002)

Abbreviations: DMBA=dimethylbenz(a)anthracene; GD=gestational day; MPA=medroxyprogesterone acetate; MNU=*N*-methyl-*n*-nitrosourea; PND=postnatal day; SPI=soy protein isolate; TEB=terminal end bud.

a Tumour models: spontaneous mammary tumour model of transgenic mice (MMTV/c-neu), and a DMBA-induced, MPA-primed mouse mammary tumour model (MPA-DMBA).

b Medroxyprogesterone acetate – priming with 2 × 20 mg pellets on week 7, DMBA 1 mg single dose orally on weeks 9, 10, 12, and 13.
